# Conformational epitope matching and prediction based on protein surface spiral features

**DOI:** 10.1186/s12864-020-07303-5

**Published:** 2021-05-31

**Authors:** Ying-Tsang Lo, Tao-Chuan Shih, Tun-Wen Pai, Li-Ping Ho, Jen-Leih Wu, Hsin-Yiu Chou

**Affiliations:** 1 Department of Computer Science and Engineering, National Taiwan Ocean University, Keelung, Taiwan; 2 Department of Computer Science and Information Engineering, National Taipei University of Technology, Taipei, Taiwan; 3 Center of Excellence for the Oceans, National Taiwan Ocean University, Keelung, Taiwan; 4 Department of Bioscience and Biotechnology, National Taiwan Ocean University, Keelung, Taiwan; 5 Institute of Cellular and Organismic Biology, Academia Sinica, Taipei, Taiwan; 6 Department of Aquaculture, College of Life Science, National Taiwan Ocean University, Keelung, Taiwan

**Keywords:** Binding region prediction, Epitope, Paratope, Conformational analysis, Spiral feature vector

## Abstract

**Background:**

A conformational epitope (CE) is composed of neighboring amino acid residues located on an antigenic protein surface structure. CEs bind their complementary paratopes in B-cell receptors and/or antibodies. An effective and efficient prediction tool for CE analysis is critical for the development of immunology-related applications, such as vaccine design and disease diagnosis.

**Results:**

We propose a novel method consisting of two sequential modules: matching and prediction. The matching module includes two main approaches. The first approach is a complete sequence search (CSS) that applies BLAST to align the sequence with all known antigen sequences. Fragments with high epitope sequence identities are identified and the predicted residues are annotated on the query structure. The second approach is a spiral vector search (SVS) that adopts a novel surface spiral feature vector for large-scale surface patch detection when queried against a comprehensive epitope database. The prediction module also contains two proposed subsystems. The first system is based on knowledge-based energy and geometrical neighboring residue contents, and the second system adopts combinatorial features, including amino acid contents and physicochemical characteristics, to formulate corresponding geometric spiral vectors and compare them with all spiral vectors from known CEs. An integrated testing dataset was generated for method evaluation, and our two searching methods effectively identified all epitope regions. The prediction results show that our proposed method outperforms previously published systems in terms of sensitivity, specificity, positive predictive value, and accuracy.

**Conclusions:**

The proposed method significantly improves the performance of traditional epitope prediction. Matching followed by prediction is an efficient and effective approach compared to predicting directly on specific surfaces containing antigenic characteristics.

## Background

A B-cell epitope, also known as an antigenic determinant, is the surface portion of an antigen that interacts with a B-cell receptor and/or an antibody to elicit either a cellular or humoral immune response [1, 2]. Because of binding specificity characteristics, B-cell epitopes possess a huge potential for immunology-related applications, such as vaccine development, drug design and disease prevention, diagnosis and treatment [3, 4]. Although clinical and biological researchers usually rely on biochemical/biophysical experiments to identify epitope-binding sites in B-cell receptors and/or antibodies, such experiments are expensive, time-consuming and not always successful [5]. Therefore, in silico methods that reliably predict B-cell epitopes could simplify immunology-related experiments [6]. By applying accurate epitope-prediction tools, immunologists can focus only on high-likelihood antigenic protein segments and reduce their experimental efforts. It was also reported that computational methods could significantly reduce the epitope prediction time and costs of vaccine development [7–9].

In general, epitopes are categorized into linear (continuous) and conformational (discontinuous) types [10–12]. A linear epitope (LE) is a short, continuous sequence of amino acids located on the surface of an antigen. Although an isolated LE lacks conformational information, it is usually flexible and can adapt its conformation to form weak interactions with a complementary antibody. Many researchers have focused on LE prediction, and a number of LE prediction systems have been developed with some accuracy. These systems require only a protein sequence as a query input, and well-known systems include BEPITOPE [13], BCEPred [14], BepiPred [15], ABCpred [16], LEPS [17, 18], and BCPreds [19]. The algorithms calculate physicochemical properties, such as polarity, charge or secondary structure of residues within the targeted protein sequences, and then apply quantitative matrix analyses or machine-learning algorithms, such as a hidden Markov model, support vector machine or artificial neural network, to predict LEs.

The second type of epitope, a conformational epitope (CE), is composed of residues that are not continuous in sequence, but are rather adjacent on the structural surface of the protein after folding [20]. The majority of B-cell epitopes are CEs, and the number of CEs on native proteins has been estimated to be ~ 90% of all B-cell epitopes [21, 22]. Research focusing on the identification of CEs has provided practical and valuable results. The first 3D CE prediction system, CEP, was developed in 2005 [23], and nearly twenty other CE prediction systems or algorithms have been developed in the past decade.

Following from the chronological progress of prediction technologies, CE prediction technologies can be divided into four categories. All published CE epitope prediction systems and corresponding algorithms are listed in Table 1. The first category applies statistical approaches to identify high-propensity epitope features on antigen proteins or designs classifiers based on a combination of weighted epitope features. Examples include: the CEP server, which was developed based on accessibility of amino acid residues [23]; Discotope, which integrates surface/solvent accessibility, contact numbers, and amino acid propensity scores [22, 24]; BEPro, formerly known as PEPITO, which utilizes amino acid propensity scores, solvent accessibility and side-chain orientations quantified by half-sphere exposure in a linear regression [25]; PEPOP, which uses accessible surface residues and segments from putative discontinuous epitopes to predict discontinuous B-cell epitopes [26]; SEPPA server, which combines propensity scores of unit patches of residue triangles, amino acid propensity and clustering coefficients [27]; ElliPro, which applies protrusion index (PI) features to protein surface protruding areas [28]; and EPCES, which implements prediction methods using residue epitope propensity, conservation score, side chain energy score, contact number, surface planarity score and secondary structure composition [29].

**Table 1:** Conformational epitope prediction system or algorithm analysis

System name	Instructions	year	published	Features and method	Feature classify
CEP	Web system http://196.1.114.49/cgi-bin/cep.pl	2005	Nucleic Acids Research	relative solvent accessibility	Physical
DiscoTope 1.0	Web system http://tools.iedb.org/stools/discotope/discotope.do	2006	Protein Science	Amino acid log-odds and contact numbers	Physical
Rapberger’s	Algorithm	2007	Journal of Molecular Recognition	solvent accessibility, shape complementarity and binding energies	Physical +Chemical
BEpro (PEPITO)	Web system http://pepito.proteomics.ics.uci.edu	2008	Bioinformatics	DiscoTope features and side chain direction RSA and HSE	Physical
ElliPro	Web system http://tools.immuneepitope.org/ellipro	2008	BMC Bioinformatics	Protrusion index (Elliptical surface)	Physical
PEPOP	http://diagtools.sysdiag.cnrs.fr/PEPOP/	2008	BMC Bioinformatics	ASA and epitope sequence	Physical
SEPPA	Web system http://lifecenter.sgst.cn/seppa/	2009	Nucleic Acids Research	Amino acid propensity, clustering coefficient, ASA	Physical +Chemical +Triangulation
Epitopia	Web system http://epitopia.tau.ac.il	2009	BMC Bioinformatics	44 structure features and 41sequence feature with Naïve Bayes classifier	Physical +Chemical + machine learning
EPCES	Web system http://sysbio.unl.edu/EPCES/	2009	BMC Bioinformatics	residue epitope propensity, conservation score, sidechain energy score, contact number, surface planarity score, and secondary structure composition.	Physical +Chemical
Soga’s	Algorithm	2010	Protein Engineering	Amino acid propensity, ASEP	Physical
Bepar	Algorithm	2010	BMC Structural Biology	paratope-epitope interacting biclique and cooccurrent pattern of interacting residue pairs	Physical +Antibody info.
CBTOPE	Web system http://osddlinux.osdd.net/raghava/cbtope/submit.php	2010	Immunome Research	Binary profile of patterns (BPP) + Physico-chemical profile of patterns (PPP) + Composition profile of patterns (CPP) with SVM	Physical +Chemical
EPSVR	Web system http://sysbio.unl.edu/services/	2010	BMC Bioinformatics	EPCES feature with SVR	Physical +Chemical + machine learning
EPMeta	Software	2010	BMC Bioinformatics	Combine EPSVR and others 5 system	Multiple system
Bpredictor	Software	2011	BMC Bioinformatics	thick surface patch and amino acid frequency with random forest (RF) algorithm	Physical + machine learning
Liu’s	Algorithm	2011	Journal of Proteomics & Bioinformatics	relative solvent accessibility and b factor with logistic regression	Physical
ABepar	http://155.69.2.25/~s080011/index.html	2011	Computational Biology Bioinformatics	Amino Acid pair and contact residue pairs with HMM	Physical + Antibody info. + machine learning
DiscoTope 2.0	Web system www.cbs.dtu.dk/services/DiscoTope/	2012	PLOS ONE	Amino Acid pair and RSA	Physical + different host
Wen Zhang’s	http://bcell.whu.edu.cn	2012	PLOS ONE	Combine 6 CE systems and 4 LE systems	Multiple system
PatchTope	http://www.fci.cu.edu.eg:8080/PatchTope/	2012	American Journal of Bioinformatics Research	Surface patch for RSA and b factor with SVM	Physical +Chemical + machine learning
CE-KEG	Web system http://cekeg.cs.ntou.edu.tw	2013	BMC Bioinformatics	Energy and amino acid pair	Physical +Chemical
SEPPA2.0	Web system http://lifecenter.sgst.cn/seppa2/	2014	Nucleic Acids Research	RSA, clustering coefficient, ASA, AAindex for ANN with logistic regression	Physical +Chemical
EpiPred	Web system http://opig.stats.ox.ac.uk/webapps/sabdab-sabpred/EpiPred.php	2014	Structure Bioinformatics	geometric fitting and knowledge-based asymmetric antibody-antigen scoring,then using docking program to enhance prediction ability	Physical +Chemical +docking program
Hu’s	Algorithm	2014	BMC Bioinformatics	Combine 4 CE systems and 4 LE systems	Multiple system
CBEP	http://59.73.198.144:8088/CBEP/	2014	BioMed Research International	evolutionary profile, secondary structure, disorder zone, dipeptide composition and physicochemical properties with multiple ML	Multiple system + multiple machine learning
CeePre	Algorithm	2014	BMC Bioinformatics	B factor, Evolutionary, Amino acid log-odds with random forest	Physical +Chemical +machine learning
PEASE	Web system http://www.ofranlab.org/PEASE	2015	Structural bioinformatics	surface accessibility, secondary structure, predicted disorder, predicted interaction hotspots, the amino acid considered, and amino acids neighboring in sequence with Random Forest	Physical + machine learning + Antibody info.
Sun’s	Algorithm	2015	Bio Research International	EPCES features and mimotope knowledge to enhance prediction ability	Physical +chemical + mimotope
PUPre	Algorithm	2015	BMC Bioinformatics	209 features +PU learning	Physical +Chemical + machine learning
SePre	Algorithm	2017	BMC Genomics	239 features + two staged heterogenous learning method	Physical +Chemical + multiple machine learning
SEPIa	Algorithm	2017	BMC Bioinformatics	13 sequence-based features with naïve Bayesian and random forest classifier	Physical +Chemical + multiple machine learning

The second category uses machine learning methods to predict CE epitopes. For example: Epitopia, which employs 44 structure features and 41 sequence features within a Naïve Bayes classifier [30]; EPSVR, which utilizes six epitope characteristics of the EPCES method and combined support vector regression techniques [31]; Bpredictor, which was constructed using thick surface patches and amino acid frequencies in a random forest model [32]; ABepar, which employs amino acid pairs and contact residue pairs within a hidden Markov model [33]; SEPPA2.0, which enhances prediction performance of previous systems by using accessible surface area, relative accessible surface area, clustering coefficient and AAindex in an artificial neural network with logistic regression [34]; and CeePre, which uses B factor, evolutionary conservation and amino acid log-odds to build a random forest learning model [35].

The third category applies multiple system prediction, also known as ensemble learning or multiple layer prediction. For example: EPMeta, which integrates EPSVR and five other existing prediction servers (EPCES, EPITOPIA, SEPPA, PEPITO, and DiscoTope1.2) to provide consensus prediction results [31]; Zhang et al., which proposes a prediction method by combining six CE prediction systems and four LE prediction systems [36]; Hu et al., which integrates four CE prediction systems and four LE prediction systems to perform a multiple layer prediction [37]; and SEPIa, which proposes a prediction method by combining a Naïve Bayes classifier and a random forest classifier [38].

The fourth category combines additional information to enhance prediction accuracy. This group includes: EpiPred, which employs a protein-docking program to assist in discontinuous epitope prediction [39]; Bepar [40] and PEASE [41], which require antibody sequences from users for CE prediction; and Sun et al. integrated mimotope analysis to increase prediction accuracy [42].

Although a large number of CE prediction systems were published, the performance of B-cell epitope prediction systems thus far is not satisfactory. The literature has suggested several reasons why CE prediction techniques have not achieved satisfactory performance [43–47]: (1) Compared to the variety of antigen-antibody complexes existing in nature, the collected epitope dataset is still too small and inconsistent. (2) Non-epitope amino acids are frequently defined as antigenic epitopes. The true epitopes may possess only a few critical surface residues, but researchers often define misidentified adjacent amino acids as epitopes. (3) It is difficult to evaluate the true prediction performance of different systems. Due to each system using their own training and testing datasets, there is no benchmark standard for a fair evaluation. (4) True undetected antigen epitopes are being treated as non-antigenic epitope regions. In addition, geometric structural information could provide more useful characteristics than sequences for unknown antigenic epitope prediction. However, in recent years, most of the CE prediction tools have applied similar characteristics for constructing classifiers and prediction systems and no new critical or effective identification features have been found. Only transformations of a variety of prediction technologies in the field of machine learning and adjustment of training/testing datasets to its best prediction results have been reported.

**Fig. 1:**
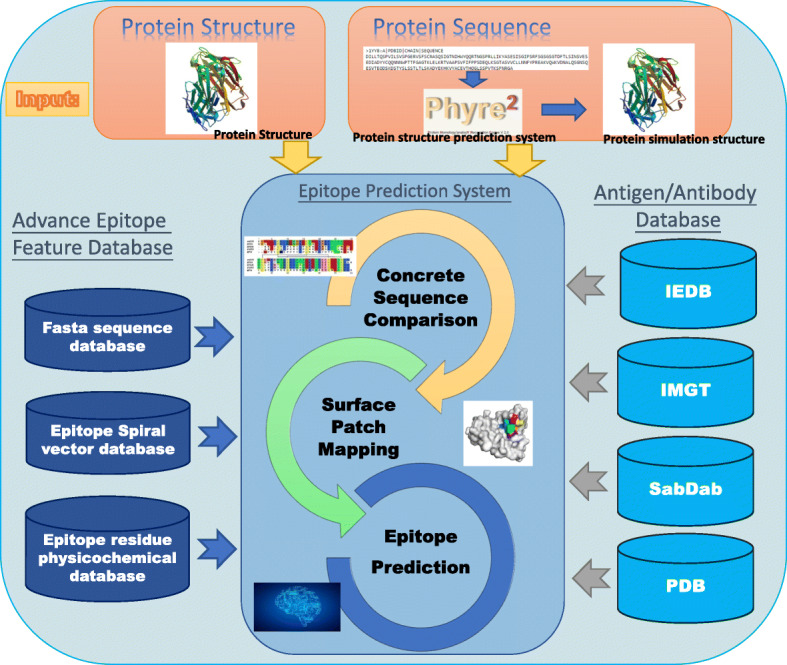
A flowchart of our conformational epitope prediction system

In this paper, we started from the perspective of vaccine developers and drug designers. The main goal was to propose a discontinuous epitope search and prediction system with the central concept of “matching first, and prediction second”. The schematic diagram of our designed system is shown in Fig. 1. A query protein sequence/structure is uploaded to the system and it automatically matches all previously-published epitope regions. When the query protein possesses only sequence information, the system automatically transfers the sequence to Phyre2 webserver [48] to generate a simulated protein structure. The designed system searches the most established epitope databases, such as IEDB [49], IMGT [50], SabDab [51], and PDB [52], to find any identical or highly similar antigenic epitopes. If the query object possesses similar antigenic epitopes within the databases, the system defines the mapped regions as candidate antigenic epitope regions. Otherwise, if the matching process cannot find any similar epitope sequences or structures from the databases, the antigen epitope prediction module will be activated. The designed system directly displays all similar antigen protein structures and corresponding antibodies and provides links to additional related resources for downstream applications. In summary, two searching methods (sequence matching and surface patch matching) and two predictive methods (CEKEG [53] and SFVP [54]) were integrated for a comprehensive CE prediction system. The integrated system can produce a variety of mapped and predicted antigenic epitopes through efficient and effective search and prediction algorithms.

## Methods

### Sequence-based epitope search

The BLAST algorithm is widely used as a sequence comparison tool for matching similar or identical sequences. Many antigenic database systems provide similar antigen search services, such as AntigenDB [55] and SabDab [56]. These are useful only for query antigens with corresponding amino acid fragments available, but without resolved corresponding 3D protein structures. In this study, we collected 1694 sequences from the IEDB database as an initial target database. Since the PDB file format has been verified and manually curated by protein crystallographers, there are many modifications, such as amino acid insertion, deletion, starting position, multiple model records, and multiple positions of residues. However, the corresponding protein sequences in FASTA format only contain the sequential order of amino acids within a protein. Therefore, in order to correctly map the amino acid number to the known antigenic sequence searched by BLAST, we prepared a structure-sequence correspondence look-up table for antigen epitope residues. Using this table, the residue number within the PDB file and the corresponding FASTA sequence could be mapped appropriately.

**Fig. 2:**
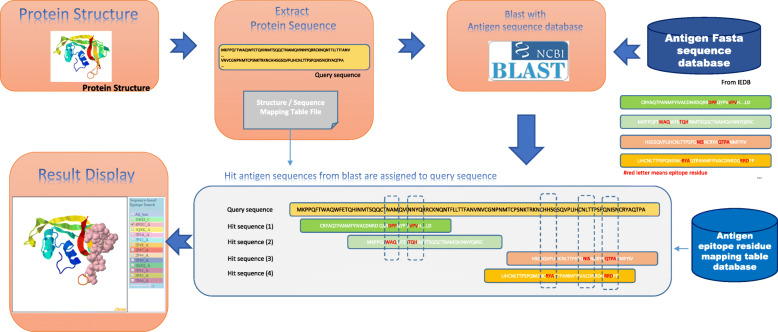
A flowchart of the sequence-based antigen epitope search method

This proposed system acquires a query protein structure as the input for analyzing its antigenic epitopes. The corresponding sequence from the PDB file format is extracted and saved as a FASTA file. Then, BLAST+ is applied to the query protein for matching to similar sequences from the previously-collected known antigen database. Finally, the JSmol protein structure molecular viewer is used to display the mapped results. The system shows each mapped known antigenic epitope residue and its corresponding position in the query structure. In addition, the system displays all relevant information about the known antigens, such as antigenic type, antibody/antigenic domain, antigen epitope/antibody paratope residue mapping table, antigenic name, host/antigen/antibody species name and corresponding links to other antigen-antibody databases. The flow chart is shown in Fig. 2.

### Surface-based epitope search

The BLAST tool provides direct searching of a known antigenic sequence database, and the matched antigen segments possess identified epitopes which could be considered as the reference epitope segments for the query sequence. However, the number of antigen-antibody binding pairs in nature is greater than the epitopes collected from all existing antibody databases. In addition, when an antibody binds to a specific antigenic epitope, it is thought that the surface residue characteristics at the binding regions are strongly related to the structural conformation of the binding regions, binding affinity and specificity. If the query protein sequence is not more than partially similar to the sequences collected in the database, the sequence-based approach would fail to discover any possible antigenic epitope. Therefore, we also propose a surface-structure-based approach to complement our sequence-based approach by employing surface spiral vector matching analysis. Each individual surface residue on the query antigen structure is used to formulate a corresponding surface spiral vector, and the calculated surface spiral vectors are compared to all previously-known antigenic epitope spiral vectors. A surface spiral vector of a residue located on a protein structural surface is defined as a sequential residue sequence containing all adjacent surface residues within a defined radius. The sequential order of all associated neighboring residues among the corresponding spiral vector is constructed by a shortest distance path approach and formulated as a non-repeated shortest circle path. Hence, a surface residue could be identified as a candidate epitope residue if high antigenic affinity and similarity are verified by comparing the corresponding spiral vectors of the query residue and all previously-known antigenic epitope residues. After performing the spiral vector matching process, all candidate antigenic epitope residues are integrated as a CE by evaluating their 3D geometrical distances, and finally, the system reveals all possible grouped epitope regions that could be bound with a specific antibody.

The spiral vector searching process utilizes the following steps:

- Surface spiral vector generation

To create a surface spiral vector of a selected surface residue, first the adjacent residues are identified. Here, we used the MSMS program to create a triangular-mesh of the surface of the query antigen structure. This process obtains all the adjacent residues of each surface residue. However, these neighboring residues are not arranged or listed in a clockwise or counterclockwise direction. In order to create a corresponding spiral vector sequence of the surface residues, we calculated the shortest distance as the space neighboring distance between all pairs of neighboring surface residues, considering the related surface atoms belonging to the two adjacent residues. Then, a group of mutual distances obtained from the neighboring surface residue pairs was applied to construct a circle of surface amino acids. This forms the shortest distance problem and can be converted into the fairly well-known Traveling Salesman Problem [57]. Using either heuristic approaches or dynamic programming method to find a non-repeated shortest circle path, we can obtain a corresponding geometric vector for each surface residue and apply this surface spiral vector for surface matching. The pseudocodes of identifying the corresponding spiral vector of a surface residue through heuristic approaches are written as the following,

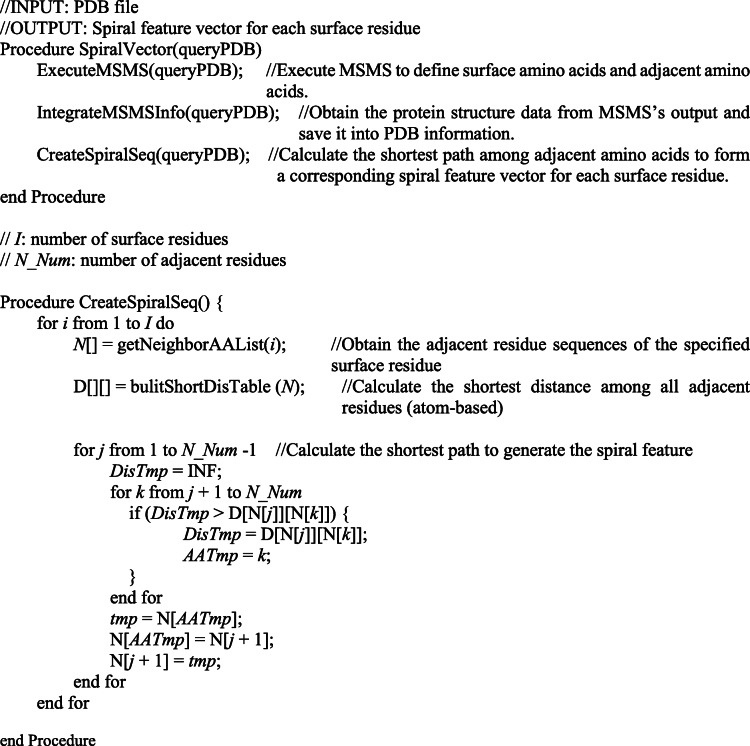



**Fig. 3:**
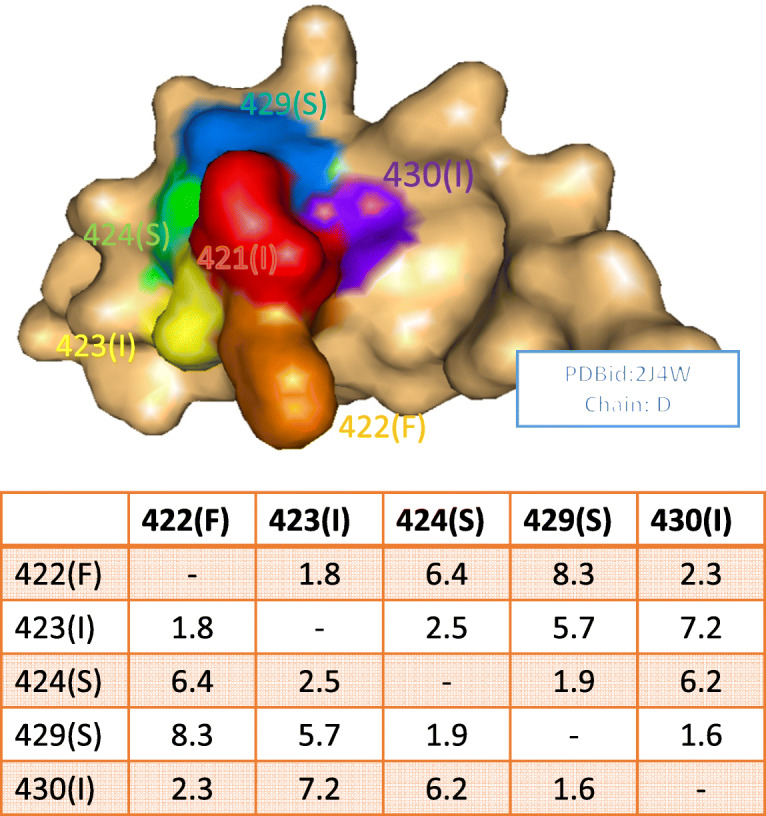
Example of a surface spiral vector. **a** A simple illustration of a spiral vector. **b** The shortest distance table for a group of neighboring surface residues

To illustrate the calculation of a spiral vector using a simple example (Fig. 3 (a)), we selected the residue number 421 (isoleucine, Ile) from the functional domain D of protein 2F4W. Using the MSMS program to perform protein surface identification, we can obtain five adjacent residues for the query residue 421, which are 422, 423, 424, 429 and 430. The system then automatically calculates the shortest distances between all surface residue pairs as shown in Fig. 3 (a), and the five adjacent amino acids are sequentially enumerated for all possible circular permutations (2^5^ = 32 cases). After calculating all possible circular distances, the sequence of 422(F) - > 423(I) - > 424(S) - > 429(S) - > 430(I) - > 422(F) is obtained as the spiral feature with the shortest distance. Hence, the sequence residue pattern of “F-I-S-S-I” is the spiral vector for the central residue of 421, and the residue pattern of “I-S-S-I-F” is its inverse spiral vector.

b) Spiral vector comparison for known epitopes

A total of 20,565 antigenic epitope residues from 1694 sequences in IEDB were used to calculate their corresponding surface spiral vectors, and a surface spiral vector database was constructed for the following BLAST approach. To match all spiral vectors derived from surface residues of a query protein, the BLASTp-short tool is applied to find matches in the constructed target spiral vector database. A single surface residue is identified as a candidate epitope residue if its corresponding spiral vector is similar or identical to the vectors of known antigenic epitopes. Since the surface spiral vector features are constructed without consideration of clockwise or counterclockwise order, we perform an additional searching process using the inverse order of the query vector against the spiral vector database.

**Fig. 4:**
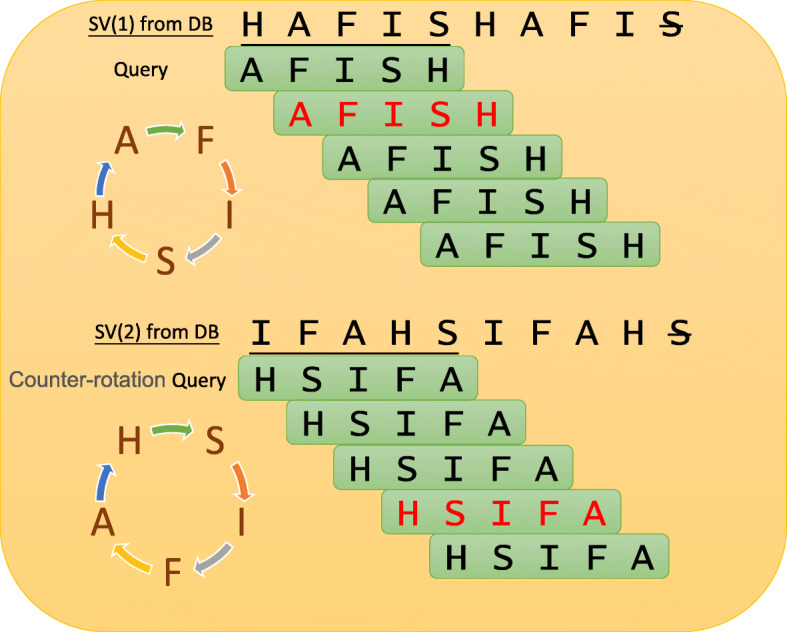
An example of spiral feature vector querying for the spiral vector sequence “A-F-I-S-H” and its reversed pattern “H-S-I-F-A” against two known epitope spiral vectors, “H-A-F-I-S” and “I-F-A-H-S”

Due to the non-directional and rotational characteristics of the spiral feature vectors, all possible rotational patterns of a spiral vector must be tested, which will increase computational time. Here, we designed a simple method to accelerate searching performance by head-to-tail tandem repeat known antigenic epitopes. For example, if an original known spiral sequence was “A-R-G-F”, we extended it repeatedly to a new pattern of “A-R-G-F-A-R-G-F”. Thus, when the system applies the BLASTp-short tool for short sequence searches, it will increase successful matching rates with all known spiral vectors even if the query pattern was rotated or shifted. In addition, the system provides a parameter for removing unreasonable search results by validating the pattern length less than a certain percentage of spiral feature vectors of known epitopes. Here we applied 50% as a default setting since we repeated all known antigenic epitopes in previous spiral feature vector preparation. This filtering processes could avoid the occurrence of a query sequence completely matched with a repeated spiral feature vector. It should be noticed that an extended and repeated spiral feature vector is for fast matching procedure, but not a true epitope. An example is shown in Fig. 4 and is described below.

As shown in Fig. 4, if a user wants to compare two spiral vector pairs of (“A-F-I-S-H, H-A-F-I-S”) and (“A-F-I-S-H, “I-F-A-H-S”), we must fix one sequence first and rotate the other spiral sequence feature for the best alignment. In order to solve the problem of an undefined initial residue within a circular feature vector, we extend the original spiral feature vectors of known antigenic epitopes by repeating the vector twice and subtracting the last amino acid. Therefore, the sequence searching method only needs to scan the query sequence once for similarity verification. In addition, since it is not known in advance if established spiral vectors were formulated in a clockwise or counterclockwise direction during feature construction, the query sequence (“A-F-I-S-H”) and its reverse pattern (“H-S-I-F-A”) should be processed simultaneously to ensure a comprehensive comparison to the epitope spiral feature database. In this way, the query surface amino acid group will be compared to the adjacent amino acid groups of known antigenic epitopes in different circular directions.

iii) Identification of high-potential antigenic residues

**Fig. 5:**
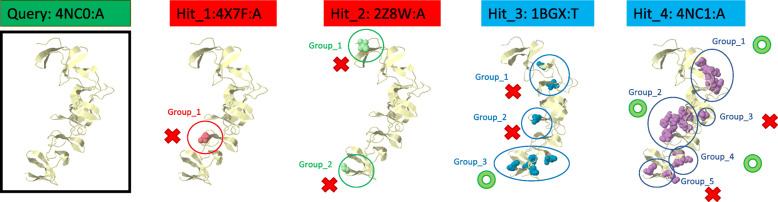
A protein structure (PDBid: 4NC0) is applied as a query structure. The system first returns similar known antigenic structures through the surface search method. Red crosses represent the predicted surface residues which were discarded due to a small number of neighboring residues predicted as candidate epitope residues. The green circles represent clustered predicted surface epitope residues based on the distance threshold settings

After comparing all surface spiral feature vectors of a query protein to all known spiral vectors derived from known antigenic epitopes, surface residues possessing high-potential antigenic epitope characteristics are revealed and annotated. However, the system will automatically remove the identified surface residues which were matched by coincidence. To achieve this goal, the system collects all matched surface residues possessing similar spiral vectors obtained from known antigenic residues, and then calculates the geometric distance of each pair of matched surface residues. The distance is defined and calculated using the previously-described method for constructing spiral features, which defines the shortest distance as the distance of the two closest surface atoms of the two selected residues. A recursive method is performed for grouping high-potential antigenic residues according to their surface amino acid distance. The number of surface amino acids in each clustered group is discarded if it is less than a threshold setting. It is observed that clustered groups possessing similar spiral characteristics have closer distances. As an example, the functional domain A of the 4NCO protein structure in Fig. 5 was used as the query protein for matching similar protein surface patches collected from the antigen database. Through spiral feature vector comparison, four known antigenic epitopes were identified. After grouping high-potential antigenic amino acids by their spatial distance attributes, each known antigen was assigned to one or more groups. Finally, the system automatically deleted certain groups when the number of matched amino acids was less than a threshold setting. For the example shown in Fig. 5, the result shows that only Group_3 from 1BGX_T and Group_1, Group_2, Group_4 from 4NC1_A were selected and displayed as the matched epitopes for the query protein.

iv) Continuous surface patch formation by anchor extension

Although high-potential antigenic residues, called as anchors, are identified and clustered based on matching spiral feature vectors, these antigenic epitopes might be dispersed and discontinuously located on a protein surface due to low evolutionary conservation. It is therefore necessary to stitch adjacent surface residues to form a continuous surface patch using an automatic procedure. Here, we define a fixed radius as an extension region for grouping identified anchors. After each identified anchor is expanded outward by the default radius, the overlap of two adjacent anchors can be analyzed. In other words, when a residue is covered by at least two disks of identified anchors, the surface residue is additionally selected and considered as an extended group of identified epitope residues. All grouped anchors and extended epitope residues together form a complete and contiguous epitope patch.

**Fig. 6:**
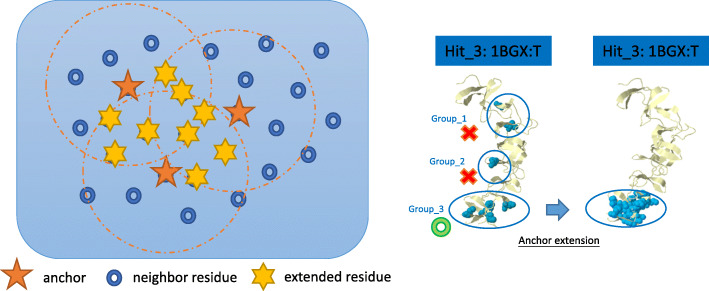
An example of anchor extension. (Left) The process of anchor extension. (Right) Dispersed anchor points connect through adjacent residues to form a surface patch

In Fig. 6 we present the concept of grouping anchors and extending neighboring residues. The orange stars represent identified high-potential antigenic amino acids (also called as ‘anchors’), and the orange dotted lines represent disks for identifying extended residues. Under the default expansion radius setting, the adjacent surface residues for each anchor are defined and all extended residues are retrieved to form a complete surface patch. It can be observed in Fig. 6 (right) that only Group_3 of the Hit_3: 1BGX could be extended to a continuous surface patch.

### Searching method

**Fig. 7:**
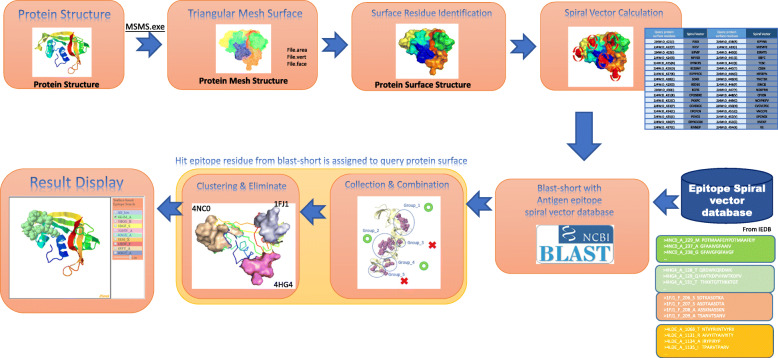
A flowchart of the surface-based antigen epitope search method

In order to efficiently search all possible epitope regions through surface patch matching, we developed an epitope search algorithm for fast comparison and visualization. The main purpose of the algorithm is to treat all surface residues as individual objects. Each individual amino acid has its corresponding spiral sequence feature vector constructed based on the adjacent amino acids, and this vector is compared to the spiral vectors of known epitopes to identify surface patch similarities. After extraction, collection, combination, clustering and elimination, the identified surface patches are identified. The detailed flow chart is shown in Fig. 7. The designed system allows users to upload a protein structure or a PDB code to discover all possible epitope regions. When a user chooses to analyze a protein structure by PDB code, the system will automatically connect to the RDSB PDB website to download its corresponding structural information. The user can specify one or more functional domains as the search terms, the system retrieves all specified functional domains. After the application of MSMS software for triangular meshing, the original PDB file format with atomic type and three-dimensional spatial coordinates is converted into a series of simulated protein structure files that represent the protein surface structure. The system produces a set of data files with extensions of .vert, .face, and .area. The generated files also contain information on the coordinates of each triangle point, any three points formed by the surface, and the triangle grid area. From the .vert and .face files, the system can acquire any surface amino acid and its adjacent amino acids list, and the .area file defines the surface residues of the query protein.

Corresponding surface spiral feature vectors of all surface amino acids of the query protein are calculated according to the methods described above. The BLASTp-short program is applied to search similar epitope spiral vectors from the constructed database by querying all surface spiral vectors of the query protein in both clockwise and counterclockwise directions. BLASTp-short differs from BLAST in its ability to search short sequences. The mapped results from BLASTp-short are collected and integrated according to different antigenic protein structures. The default distance thresholding parameter for clustering associated residues is 15 Å. When the distance between any two residues is greater than the default setting, the two residues are classified into different groups. A recursive clustering algorithm is used to find all possible binding areas. In order to exclude clustered groups with small numbers of similar spiral feature vectors, our designed system removes clustered groups less than 4 amino acids. Finally, all the resulting high-potential antigenic clusters and their corresponding antigenic epitope amino acids are displayed. All detail information of the proposed system and more illustrated examples can be found in Lo’s PhD thesis [58].

### Constructing the verification dataset

**Fig. 8:**
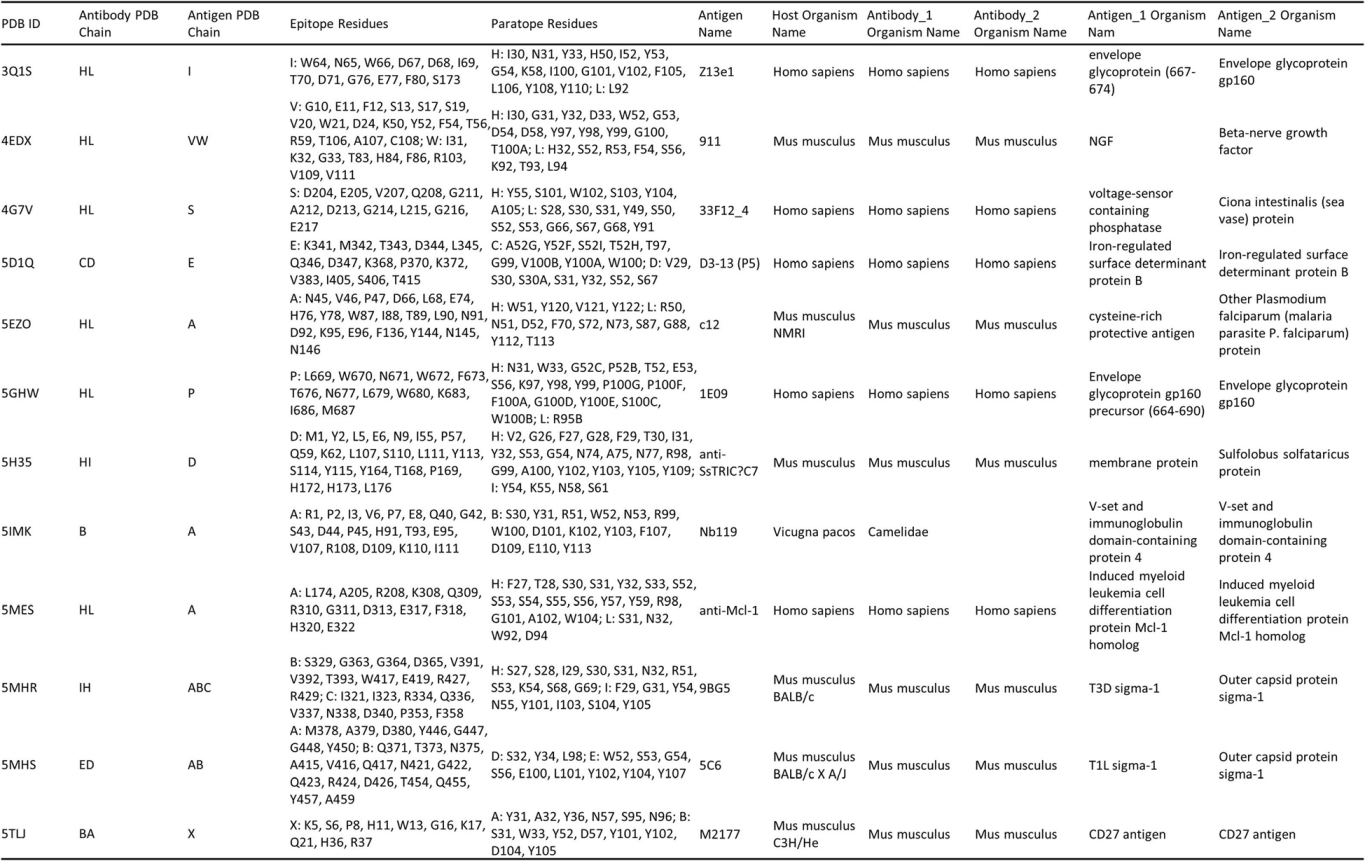
A detailed description of proteins in the validation set

In order to objectively evaluate the performance of our prediction system, we utilized a set of exclusive antigen-antibody complexes from a previously-collected epitope database. From the IEDB, we collected a set of 90 newly-reported and labelled antigen-antibody complexes, several of which were similar in structure or identical in sequence to the previously-collected epitope sequence database. When considering a minimum sensitivity of 50% as a successful prediction, a total of 42 antigenic proteins were correctly predicted by CSS, and 48 by SVS. If the threshold setting was reduced to a sensitivity of 25%, a total of 50 correct predictions were achieved by CSS, and 76 by SVS. This clearly shows that query structures possessing similar sequences or surface patches within the known epitope database can easily be identified. Next, we analyzed the failed matching results caused by low structure/sequence similarities. Sequences of the new antigenic proteins were aligned with all previously collected antigens by the BLAST algorithm, and we excluded antigens with E-values less than 1e-10. A total of 29 protein structures with low sequence similarities remained after comparison to all previously-known epitope sequences. The CD-HIT tool [59] was then used to cluster the 29 sequences according to results of a pairwise sequence alignment. Sequences with similarities greater than 70% were clustered, and only one representative structure from each group was selected as the group representative in the following analysis. As a result, only 12 representative structures with differential sequence contents were selected for validating the proposed search methods and comparing them to existing methods (Fig. 8).

## Results

In this study, we have adopted 12 newly annotated and non-redundant protein structures as a testing set for comparing our CE prediction system with all other systems available online. Although there are dozens of published prediction systems developed over the past decade, more than half of them are not maintained online or accessible. The six comparable systems we identified were ElliPro [28], Epitopia [30], EPSVR [31], CBTOPE [60], Discotope [24] and CEKEG [53]. Since the input and output of each prediction system is different, we executed and evaluated their prediction performances individually. Examples of major differences include: ElliPro and CEKEG provide multiple predicted epitopes; CBTOPE requires the antigen sequence as input and predicts a set of epitopes; Epitopia reports five levels of immunogenicity scales; EPSVR only calculates the epitope score for each residue of the query protein; and Discotope 2.0 provides input antibody structure and predicts a set of epitopes. We collected all the prediction results from these systems and compared their prediction performance. We also calculated the prediction performance of our two proposed search methods. The results are shown in Table 2. In order to fairly evaluate the ability of each search and prediction system, we applied some restrictions to the prediction results. When a system reported multiple sets of prediction results, only the first three predicted results were evaluated. All systems were initially used with their own default threshold settings, if there were any. If the system could not identify any predictive candidates initially, we adjusted the settings using objective and reasonable selections to perform CE prediction. It should be noted that because our search results were based on E-values and the number of identified anchors within a single group as the ranking factors, sometimes the prediction system does not provide three predictions exactly.

**Table 2:** Comparison of the results of all available systems and proposed search methods on the validation set. *SEN* Sensitivity, *SPE* Specificity, *PPV* Positive Predictive Value, *ACC* Accuracy, *MCC* Matthew’s Correlation Coefficient, *Avg-AUC* Average Area Under the Curve. (Accessed date: July 2017)

Prediction System	SEN	SPC	PPV	ACC	F1 score	MCC	Avg-AUC	Prediction Condition
CEKEG (2013)	**0.528**	0.786	0.292	0.775	**0.370**	**0.236**	**0.657**	Best Result from Top3 prediction
ElliPro (2008)	0.343	**0.901**	**0.357**	0.826	0.300	0.227	0.622	Best Result from Top3 prediction
CBTOPE (2010)	0.334	0.665	0.087	0.597	0.133	0.000	0.500	SVM threshold: 0.3 (default)
Epitopia (2009)	0.274	0.836	0.187	0.761	0.210	0.081	0.555	Immunogenicity Scale: 5
EPSVR (2010)	0.198	0.815	0.137	0.733	0.152	0.007	0.507	Epitope score > 80
Discotope2.0 (2012)	0.190	0.847	0.244	0.773	0.140	0.065	0.518	Threshold: −3.7 (default)
**Search System**	**TPR**	**SPC**	**PPV**	**ACC**	**F1 score**	**MCC**	**Avg-AUC**	**Prediction Condition**
CSS	0.120	**0.978**	0.132	**0.890**	0.123	0.028	0.549	Best Result from Top3 prediction
SVS	**0.473**	0.869	**0.365**	**0.853**	**0.396**	**0.307**	**0.671**	Best Result from Top3 prediction

To evaluate the performance of the proposed method at the level of the amino acid residue, four indicators were applied to measure individual performance. These indicators include sensitivity (SEN), specificity (SPE), positive predictive value (PPV), F1 score, Matthews correlation coefficient (MCC) and average area under the curve (AvgAUC). (1) SEN is defined as the percentage of true epitope residues that are correctly predicted as epitope residues; (2) SPE is defined as the percentage of non-epitopes that are correctly predicted as non-epitopes; (3) PPV is also called as precision rate which is defined as the probability that a predicted epitope is, in fact, an epitope; (4) F1 score is the harmonic average of the precision and recall rates. Precision rate is the same as PPV and recall rate is the same as SEN; (5) MCC is a measure of the predictive performance that incorporated both SEN and SPE into a single value between − 1 and + 1; (6) AvgAUC is defined as the average of SEN and SPE. These parameters are calculated with the following equations:

1 $$ \mathrm{Sensitivity}\ \left(\mathrm{SEN}\right)=\mathrm{Recall}\ \mathrm{Rate}=\frac{\mathrm{TP}}{\mathrm{TP}+\mathrm{FN}} $$

2 $$ \mathrm{Specificity}\ \left(\mathrm{SPE}\right)=\frac{\mathrm{TN}}{\mathrm{TN}+\mathrm{FP}} $$

3 $$ \mathrm{Positive}\ \mathrm{Predictive}\ \mathrm{Value}\ \left(\mathrm{PPV}\right)=\mathrm{Precision}\ \mathrm{Rate}=\frac{\mathrm{TP}}{\mathrm{TP}+\mathrm{FP}} $$

4 $$ \mathrm{F}1\ \mathrm{score}=2\mathrm{x}\frac{\mathrm{Precision}\ \mathrm{x}\ \mathrm{Recall}}{\mathrm{Precision}+\mathrm{Recall}} $$

5 $$ \mathrm{MCC}=\frac{\mathrm{TPxTN}-\mathrm{FPxFN}}{\sqrt{\left( TP+ FP\right)\left( TP+ FN\right)\left( TN+ FP\right)\left( TN+ FN\right)}} $$

6 $$ \mathrm{AvgAUC}=\frac{\mathrm{SEN}+\mathrm{SPE}}{2} $$

where TP represented the true positive; TN, the true negative; FP, the false positive; and FN, the false negative.

Our results are presented in Table 2, where the best prediction performances are in boldface and boxed and the second-best prediction performances are in boldface and underlined. It can be clearly seen that the tools using a single set of predictive systems, such as CBTOPE, Epitopia, EPSVR, or Discotope 2.0, are low in sensitivity and accuracy. As a result of the averaged performance, our proposed SVS searching method achieved the best performance in terms of PPV, F1 score, MCC and AvgAUC. Most of the second-ranking predictions (in F1 score, MCC and AvgAUC) were achieved by our previously-developed prediction system CEKEG, which also obtained the best TPR prediction indicator. The ElliPro prediction system is provided by IEDB, and had high SPE and PPV due to its conservative prediction ability. Overall, these results clearly show that our proposed searching systems outperform all other existing approaches tested.

Finally, we compared our two proposed searching methods to each other. If we set the threshold for successful identification of the 12 novel query structures as a SEN ≥ 25%, the CSS approach worked for only two structures, but the SVS worked for 9. This result indicates that if the query sequences are not similar to the epitope database, the SVS surface features comparison outperforms the CSS approach. The corresponding average AUC for prediction performances is shown in Table 3. Since the CSS method did not ever align the query to any sequence from the published epitope database, its sensitivity is low and its specificity is relatively high. In contrast, using the SVS approach to compare the surface patches and to consider the first three clusters provides a better search performance.

**Table 3:** Comparison of the CSS and SVS search methods. Calculations were performed on the testing dataset of 12 proteins. Numbers in boldface indicate the better performance for each parameter setting

Search Method	Sensitivity	Specificity	Average-AUC
CSS	0.120	**0.978**	0.549
SVS	**0.473**	0.869	**0.671**

## Conclusions

One of the most challenging research topics in developing application software for computational immunology is correctly predicting B-cell epitopes on antigenic protein structural surfaces. Although there is a long research history for both LE and CE prediction, the prediction systems are still far from producing ideal solutions. In particular, several systems developed for predicting CEs from the past few years could neither reach high-accuracy performance, nor efficient simulation. Therefore, an effective and efficient prediction tool for epitope analysis is necessary for the growth and development of immunology-related applications, such as vaccine design, drug design and disease prevention. With the rapidly increasing number of solved protein structures, CE prediction has become a necessary tool prior to wet lab biomedical and immunological experiments. In this paper, we present two major contributions to CE prediction. First, two antigen epitope search methods, CSS and SVS, were proposed. Secondly, two discontinuous epitope prediction systems, CEKEG and SFVP, were designed. We propose a novel concept of combining sequence and surface patch matching for CE prediction. In this comprehensive computation analysis, if the query structure lacks any existing homologous proteins in the database, epitope prediction will be performed.

To search for antigenic epitopes, we designed a sequential approach of matching protein sequences and surface patches to quickly find homologous antigenic epitope regions from a known epitope database. Our CSS approach facilitates searching for the most similar antigenic sequences. Our SVS approach assists to complement the shortcomings of the CSS method through matching surface spiral feature vectors to discover homologous surface patches with dissimilar and discontinuous characteristics that cannot be solved by sequence matching approaches. In addition, surface patch comparison based on spiral feature vectors does not only perform exceptionally well for matching specific antigen epitopes, but also for the unsolved problem of searching multi-structural surface patches.

To further accomplish the task of CE prediction, all possible antigenic epitope candidates are predicted using protein surface characteristics and combinatorial features of epitopes. We first designed CEKEG for CE prediction using surface energy and the frequency of amino acid pairs. In addition, we developed the SFVP system which integrates the distribution of surface amino acid content and corresponding physicochemical properties, clustering these features in different levels. A total of 57 spiral feature vectors were formulated and analyzed by a K-nearest neighbor classifier. The prediction results show that the proposed CE prediction algorithm significantly outperforms all existing prediction algorithms. Such information may facilitate the appropriate selection of initial CE anchors, forming precise CE candidates for immunological studies. Our experimental results show the superior performance of our proposed system over published computational techniques in the field of antigen-antibody interaction analysis.

Antigenic epitope prediction studies are able to assist vaccine development and drug design by significantly reducing experimental costs and time. However, CE binding region prediction has had no recent major breakthroughs in performance. Over the past decade, numerous researchers have tried to improve epitope prediction ability and the field has become increasingly aware of the high variability in binding regions to antibodies. It is certain that in the near future a larger, and more diverse, repertoire of antigen-antibody crystal complexes will be resolved. In addition, machine learning algorithms such as deep learning and AI technologies will continue to evolve through innovation. As demonstrated by our designed system, CE prediction performance can be further improved, and this will facilitate advanced applications in immuno-informatics research, vaccine design, and pharmaceutical development.

## Data Availability

Not applicable.

## Abbreviations

LE: Linear epitope
CE: Conformational epitope
CSS: Complete sequence search
SVS: Spiral vector search
SEN: Sensitivity
SPE: Specificity
PPV: Positive predictive value
MCC: Matthews correlation coefficient
AvgAUC: Average area under the curve
